# Vascular mechanisms leading to progression of mild cognitive impairment to dementia after COVID-19: Protocol and methodology of a prospective longitudinal observational study

**DOI:** 10.1371/journal.pone.0289508

**Published:** 2023-08-03

**Authors:** Cameron D. Owens, Camila Bonin Pinto, Peter Mukli, Zsofia Szarvas, Anna Peterfi, Sam Detwiler, Lauren Olay, Ann L. Olson, Guangpu Li, Veronica Galvan, Angelia C. Kirkpatrick, Priya Balasubramanian, Stefano Tarantini, Anna Csiszar, Zoltan Ungvari, Calin I. Prodan, Andriy Yabluchanskiy

**Affiliations:** 1 Oklahoma Center for Geroscience and Healthy Brain Aging, University of Oklahoma Health Sciences Center, Oklahoma City, OK, United States of America; 2 Department of Neurosurgery, Vascular Cognitive Impairment, Neurodegeneration and Healthy Brain Aging Program, University of Oklahoma Health Sciences Center, Oklahoma City, OK, United States of America; 3 Doctoral School of Basic and Translational Medicine/Departments of Public Health, International Training Program in Geroscience, Translational Medicine and Physiology, Semmelweis University, Budapest, Hungary; 4 Department of Biochemistry and Molecular Biology, University of Oklahoma Health Sciences Center, Oklahoma City, OK, United States of America; 5 Veterans Affairs Medical Center, Oklahoma City, OK, United States of America; 6 Department of Medicine, Cardiovascular Section, University of Oklahoma Health Sciences Center, Oklahoma City, OK, United States of America; 7 The Peggy and Charles Stephenson Cancer Center, University of Oklahoma Health Sciences Center, Oklahoma City, OK, United States of America; 8 Department of Health Promotion Sciences, College of Public Health, University of Oklahoma Health Sciences Center, Oklahoma City, OK, United States of America; 9 Department of Neurology, University of Oklahoma Health Sciences Center, Oklahoma City, OK, United States of America; PLoS ONE, UNITED STATES

## Abstract

**Introduction:**

Mild cognitive impairment (MCI) is a prodromal stage to dementia, affecting up to 20% of the aging population worldwide. Patients with MCI have an annual conversion rate to dementia of 15–20%. Thus, conditions that increase the conversion from MCI to dementia are of the utmost public health concern. The COVID-19 pandemic poses a significant impact on our aging population with cognitive decline as one of the leading complications following recovery from acute infection. Recent findings suggest that COVID-19 increases the conversion rate from MCI to dementia in older adults. Hence, we aim to uncover a mechanism for COVID-19 induced cognitive impairment and progression to dementia to pave the way for future therapeutic targets that may mitigate COVID-19 induced cognitive decline.

**Methodology:**

A prospective longitudinal study is conducted at the University of Oklahoma Health Sciences Center. Patients are screened in the Department of Neurology and must have a formal diagnosis of MCI, and MRI imaging prior to study enrollment. Patients who meet the inclusion criteria are enrolled and followed-up at 18-months after their first visit. Visit one and 18-month follow-up will include an integrated and cohesive battery of vascular and cognitive measurements, including peripheral endothelial function (flow-mediated dilation, laser speckle contrast imaging), retinal and cerebrovascular hemodynamics (dynamic vessel retinal analysis, functional near-infrared spectroscopy), and fluid and crystalized intelligence (NIH-Toolbox, *n*-back). Multiple logistic regression will be used for primary longitudinal data analysis to determine whether COVID-19 related impairment in neurovascular coupling and increases in white matter hyperintensity burden contribute to progression to dementia.

## 1. Introduction

Mild cognitive impairment (MCI) is an age-related neurocognitive disorder characterized by absence of dementia, and subjective and objective minimal cognitive impairments [1,2]. Recent meta-analyses of community dwelling older adults reported a 17.3% overall pooled prevalence of MCI [3], with increasing prevalence associated with age [4]. MCI is a prodromal stage to dementia [5–7] with an annual conversion rate of MCI to dementia ranging between 3–4.9% in community settings, up to 18% in specialty clinics, and 23.8% in socioeconomically disadvantaged areas [7–10]. Dementia is characterized with impairments in executive function, memory, language, and behavior, affecting up to 55 million individuals annually, markedly impairing patients quality of life and ability to perform everyday activities [11]. Significant physical, psychological, social, and economic burden is placed on patients with dementia, as well as their caregivers, families, and society at large [12,13]. Due to the immense number of individuals affected by this disease, risk factors known to increase the conversion rate of MCI to dementia (e.g., diabetes, hypertension, atherosclerosis) have enormous public health relevance [14–17].

The COVID-19 pandemic has caused significant morbidity and mortality globally, with over 103 million cases in the United States alone, as of June 1^st^, 2023. It is important to note that COVID-19 predominantly causes mortality in older adults [18–20]. Evidence suggests that COVID-19 increases the conversion rate of MCI to dementia in older individuals compared to those with no previous diagnosis of COVID-19 [21]. Acute COVID-19, and long-term effects after recovery, have been linked to a range of cerebral small vessel complications, including ischemic damage to the white matter, lacunar stroke, microhemorrhages, and cognitive dysfunction [22–24]. However, the mechanistic link between COVID-19 and the increased risk of progression from MCI to dementia remains unclear. Emerging clinical and basic science evidence suggests that SARS-CoV-2 infects endothelial cells[25], causing downstream inflammation and oxidative stress, which contributes to generalized macro- and microvascular endothelial dysfunction acutely, and following recovery of infection [26]. This dysfunction extends to the cerebrovascular circulation and has been linked to induction of cerebromicrovascular pathologies known to contribute to vascular cognitive impairment and dementia [22]. Hence, COVID-19 has detrimental effects on the cerebrovasculature, which is critical for maintaining normal cognitive function [27].

Components of the neurovascular unit, specifically the cerebromicrovascular endothelium, play a critical role in maintaining normal cognitive function, with adequate oxygen and nutrient delivery achieved through the homeostatic mechanism of neurovascular coupling (NVC) [28–31]. NVC is responsible for adjustment of local cerebral blood flow through endothelium-dependent cerebromicrovascular dilation to match the increased metabolic needs of active brain regions [32]. Preclinical evidence in aged animals demonstrate that impairment of NVC responses and cerebromicrovascular endothelial function is causally related to cognitive decline, while improvement of NVC responses via pharmacological means is associated with restored cognitive performance [33–39]. Importantly, in human trails these findings have been corroborated with cognitive impairment in aging and age-related diseases being strongly associated with impaired NVC [40–43]. However, whether deteriorating cognitive function associated with COVID-19 is causally related to impairments in NVC is unknown.

In this study, our primary aim is to establish a mechanistic link between COVID-19 and the progression of MCI to dementia. To accomplish this objective, we will utilize a comprehensive and integrated battery of vascular measurements, encompassing the quantification of small vessel ischemic damage through magnetic resonance imaging (MRI), assessment of NVC responses and endothelial function, and analysis of serum markers related to vascular disease. By examining the correlation between these measurements and COVID-19, our study aims to yield valuable insights into the underlying mechanisms that drive this progression.

## 2. Study aims and protocol

### 2.1 Study aims

1. To determine whether history of COVID-19 infection in patients with MCI disrupts systemic endothelial function and impairs NVC response compared to MCI patients with no history of COVID-19 infection.
2. To determine whether history of COVID-19 infection aggravates the impairment of fluid cognitive abilities in patients with MCI.
3. To determine whether impairment of NVC responses in recovered COVID-19 patients with MCI contributes to increased conversion of MCI to dementia.
4. To determine whether COVID-19 related increases in white matter hyperintensity (WMH) burden contribute to conversion of MCI to dementia.

### 2.2 Study design

**Fig 1:**
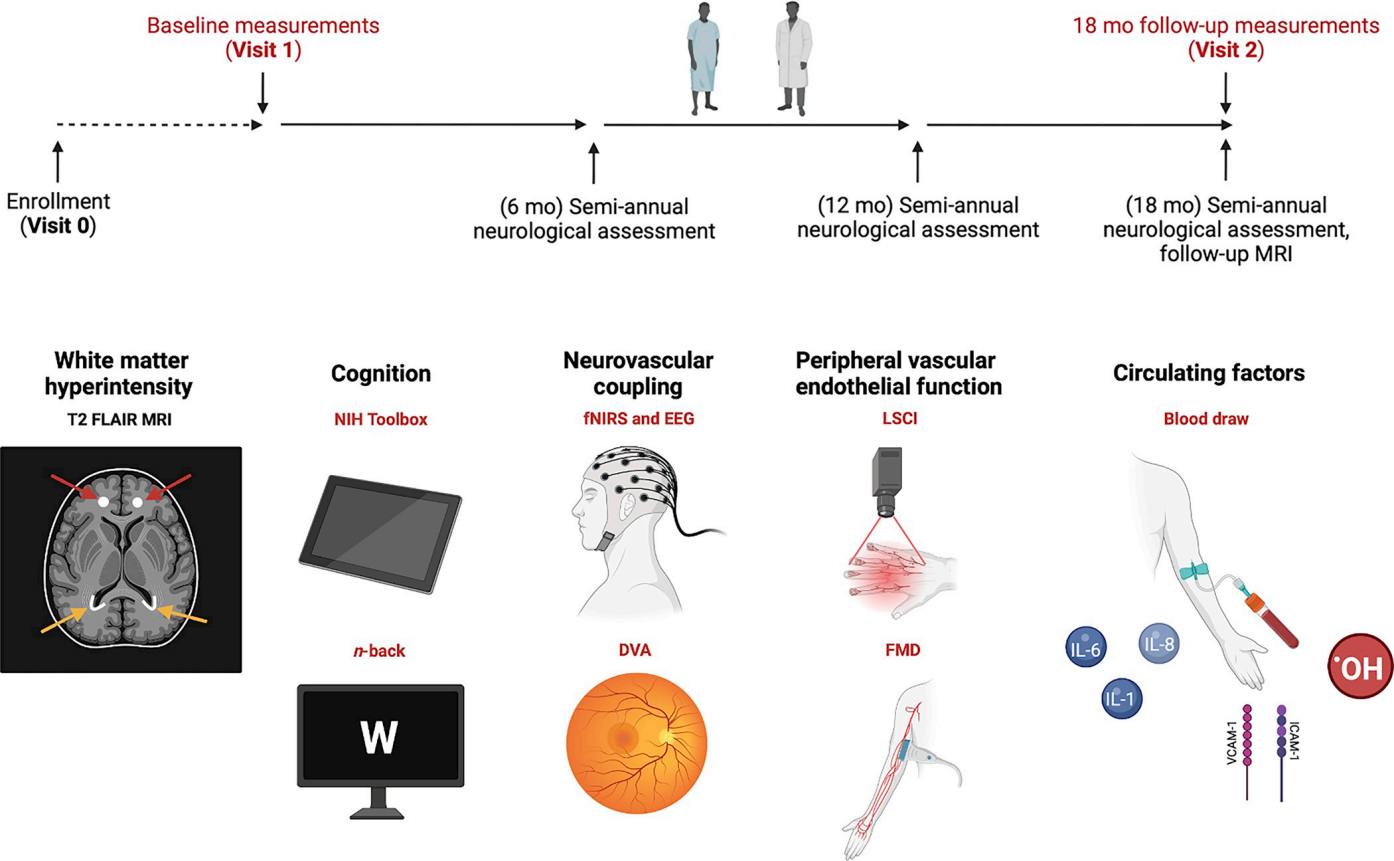
Timeline for study design and investigative measurements. (Top) Timeline of prospective longitudinal study. Visit 1 and Visit 2 (red) includes: Physiological and cognitive assessments and blood draw. (Bottom) Investigative measures including physiological and cognitive assessments and circulating factors recorded at Visit 1 and 2 (red) and baseline MRI recorded at diagnosis of MCI prior to COVID-19 pandemic, and 18-month follow-up MRI (black). Red arrows indicate deep WMH, and orange arrows indicate periventricular WMH. Abbreviations: White matter hyperintensity (WMH), Functional near-infrared spectroscopy (f-NIRS), Electroencephalography (EEG), Dynamic retinal vessel analysis (DVA), Laser speckle contrast imaging (LSCI), Flow mediated dilation (FMD).

This study will test the hypothesis that COVID-19 infection promotes systemic endothelial dysfunction, impairing NVC responses and promoting white matter damage, contributing to the progression of MCI to dementia. To test this hypothesis, we will prospectively follow patients, who are diagnosed with MCI, with or without history of SARS-CoV-2 infection, for 18 months. The timeline for study design and investigative measurements are represented in **Fig 1.** Study participants will be evaluated for eligibility and enrolled through neurology clinics at the University of Oklahoma Health Sciences Center (OUHSC) and Oklahoma City Veterans Affairs (VA) Medical Center (Visit 0). Upon enrollment, patient history, demographic data, standard risk factors for dementia, cognitive status and WMH status (MRI scan) will be recorded. Blood collection, cognitive tests (NIH-toolbox, *n-*back), and baseline physiologic assessments for peripheral endothelial function (flow-mediated dilation [FMD], laser speckle contrast imaging [LSCI]) and NVC responses (functional near-infrared spectroscopy [fNIRS], dynamic retinal vessel analysis [DVA]) will be conducted at the OUHSC Translational Geroscience Laboratory (Visit 1). Study participants will undergo semi-annual clinical neurologic evaluations as a part of standard care. In addition to COVID-19 status since enrollment (e.g., PCR+/-, symptoms), progression from MCI to dementia will be assessed by the clinical neurology team with final adjudication made through consensus management conferences. Repeat of all physiological assessments, cognitive tests, and serum collection will be performed at month 18 (Visit 2), and the second MRI scan will be performed. If some participants decide to terminate their participation in the study for any reason, they will be invited to an early termination visit, which will include the same evaluations as in Visit 2.

### 2.3 Study setting

This is an ongoing study in the Translational Geroscience Laboratory in the Department of Neurosurgery at the OUHSC in full collaboration with the OUHSC Department of Neurology and Oklahoma City VA Medical Center. The University of Oklahoma Medical Center serves the state of Oklahoma and all surrounding states, and the VA Medical Center serves up to 63,000 veterans in Oklahoma and multiple counties in Texas. Hence, patients will be evaluated for eligibility, and based on patient flow through the Department of Neurology and VA, up to 200 patients will be enrolled through our clinical collaborators in the Department of Neurology.

### 2.4 Sample size estimation

For longitudinal study design, assessing COVID-19’s effect on progression to dementia over the course of 18-months in patients with MCI, our sample size estimation is based on data from *n =* 118 patients diagnosed with MCI prior to COVID-19 pandemic. These data showed a significantly increased progression rate to dementia in MCI patients who contracted COVID-19 (10/18 patients; 55.6%) compared to patients who were never infected (23/100 patients; 23%) (*p =* 0.011) [21]. Due to continued monitoring of patients through semiannual neurologic visits as standard-of-care, we expect that dropout will not exceed the rate of 10%. Based on seroprevalence reports and total number of confirmed cases in the aging community, we anticipate that 5–15% of the control group will develop COVID-19 through the 18-month protocol. Additionally, based on the CDC 2021 national mortality data for persons aged 65 and older, accounting for COVID-19 related death, we will enroll additional participants to account for the 2–5% death per 100,000 population. Therefore, to control for these adverse events, we will require up to 200 MCI patients with and without a history of COVID-19.

### 2.5 Inclusion criteria

1. Ages between 55 and 85 years old
2. Diagnosis of MCI
3. Availability of an MRI scan before study enrollment
4. Patients with a history of COVID-19 were confirmed by PCR or serology.

### 2.6 Exclusion criteria

1. Vision or hearing impairment that would hinder the ability of participants to complete the study protocol
2. Chronic uncontrolled conditions (e.g., type 2 diabetes, hypercholesterolemia, hypertension)
3. Current cerebrovascular complications or prior complications that are clinically deemed as exclusion to the study (i.e., large vessel stroke with chronic impairment)
4. Neurodegenerative diseases (e.g., Parkinson’s, any types of dementia)
5. Eye pathologies contraindicating dynamic retinal vessel analysis (DVA): Glaucoma, shallow anterior chamber (Van Herick Grade 2–0), intraocular pressure (IOP) >21mmHg, diabetic retinopathy, distinct cataract, photosensitive epilepsy, allergy to tropicamide.
6. Medical conditions which, in the opinion of the investigative team, would render the patient inappropriate or too unstable to complete study protocol.

### 2.7 Primary outcome

The primary outcome of interest is whether COVID-19 contributes to the progression of MCI to dementia through impairment of NVC response, and promotion of white matter damage. Diagnosis of MCI and progression to dementia will be made clinically through the OUHSC Department of Neurology. Trained investigators will perform NVC measurements (fNIRS, electroencephalography [EEG], DVA) at baseline and 18 months follow-up. Burden of white matter damage (WMH) will be neuroradiologically assessed by the clinical neurology and neuroradiology team at baseline (at diagnosis of MCI prior to COVID-19 pandemic) and 18-month follow-up.

### 2.8 Secondary outcomes

- COVID-19’s effect on systemic micro- and macrovascular endothelial function (LSCI, FMD) will be measured by trained investigators at baseline and 18-month follow-up.
- Patients will be cognitively assessed by *n*-back working memory paradigm and NIH Toolbox Cognitive Battery to determine whether COVID-19 contributes to decreased fluid and/or crystalized cognitive performance. Patients will also complete select questionnaires from Emotion Battery (Emotional Support, Friendship, Loneliness, Perceived Hostility, Perceived Rejection, and Instrumental Support questionnaires) and Adverse Childhood Experience questionnaire to determine current state of social support/interaction and traumatic events during childhood, both of which are known to exacerbate and contribute to cognitive impairment [44,45]. All cognitive, emotional, and adverse childhood experience assessments will be performed at baseline and 18-month follow-up.
- Circulating levels of pro-inflammatory cytokines, antioxidant capacity, and vascular disease markers will be measured at baseline and 18-month follow-up by trained investigators to determine the long-term effects of COVID-19 on systemic inflammation, oxidative stress, and biomarkers of endothelial dysfunction. A registered nurse will perform blood draws from patients (~40 ml) and trained investigators will process blood and store in -80° freezer until ready for quantification of pro-inflammatory cytokines and vascular disease markers, and antioxidant capacity by magnetic bead-based multiplex assay and hydroxyl radical antioxidant capacity (HORAC) activity assay, respectively.

### 2.9 Study variables

All variables included in the study are represented in **Table 1**, and separated based on level of measurement (continuous, ordinal, discrete, binary). Below, the variables are described for each specific aim, as mentioned in the *Study aims* section.

**Table 1:** Summary of variables for study aims.

Variable	Method of Measurement	Operational definition	Level of measurement
**NVC**	fNIRS	Change in relative HbO concentration in cortical small vessels during cognitive task	Continuous
**Band-limited power**	EEG	Amount of electrocortical activity in distinct frequency bands during cognitive task	Continuous
**Retinal arteriolar function**	DVA	Retinal arteriolar response to flickering light stimulus (NVC stimulus)	Continuous
**Microvascular endothelial function**	LSCI	Small vessel hand perfusion following the release of 5-minute occlusion	Continuous
**Macrovascular endothelial function**	FMD	Percent change in brachial artery diameter from baseline to release of 5-minute occlusion	Continuous
**Pro-inflammatory cytokine levels**	Magnetic bead-based multiplex assay	Quantified fluorescence intensity in pro-inflammatory cytokine levels	Continuous
**Endothelial dysfunction biomarkers**	Magnetic bead-based multiplex assay	Quantified fluorescence intensity in cardiovascular disease panel markers	Continuous
**Antioxidant capacity**	HORAC activity assay	Fluorescence decay curve: time until complete decay of fluorescent probe	Continuous
**Fluid intelligence**	NIH Toolbox	Individual and categorical T score based on cognitive test responses	Continuous
**Crystalized Intelligence**	NIH Toolbox	Individual and categorical T score based on cognitive test responses	Continuous
**Emotion Score**	NIH-Toolbox	Individual T-score based on normative population data	Continuous
***n*-back working memory performance**	n-back	Percentage of correct responses and reaction time during *n*-back blocks	Continuous
**Blood pressure**	AD instruments	Blood pressure recorded during visits	Continuous
**Time since SARS-CoV-2 Infection**	Demographic	Duration from COVID-19 diagnosis to Visit 1	Continuous
**Age**	Demographic	Age (in years) of participants at each visit	Discrete
**Fazekas scale**	MRI	WMH burden (0–3) periventricular and deep	Discrete
**MoCA**	Demographic	Montreal cognitive assessment score (0–30)	Discrete
**ACE score**	ACE questionnaire	Adverse childhood experience questionnaire, scored 1–10 with a score of 10 having high ACE	Discrete
**Comorbidities**	Demographic	Past medical history and/or ongoing medically controlled for conditions	Nominal
**SARS-CoV-2 Variant**	Demographic	Estimated variant based on time (month and year) patient was infected with SARS-CoV-2	Nominal
**Vaccination History**	Demographic	Number of doses, vaccine manufacturer	Nominal
**Education**	Demographic	Highest level of education obtained	Ordinal
**Progression of MCI to dementia**	Clinical diagnosis	Use of neurocognitive screening (e.g., MoCA, clinical dementia rating) and final adjudication made through consensus management	Binary
**COVID-19 status**	*Previously confirmed COVID-19 history	COVID-19 positive or COVID-19 negative	Binary
**Sex**	Demographic	Male or female	Binary

Note. Neurovascular coupling (NVC), Functional near-infrared spectroscopy (fNIRS), Oxy-hemoglobin (HbO), Electroencephalography (EEG), Dynamic retinal vessel analysis (DVA), Laser speckle contrast imaging (LSCI), Flow mediated dilation (FMD), Hydroxyl radical antioxidant capacity assay (HORAC), White mattery hyperintensity (WMH), Montreal cognitive assessment (MoCA), Mild cognitive impairment (MCI), Adverse childhood experience (ACE).

* Indicates COVID-19 was confirmed by PCR or serologic testing.

■ Aim 1 variables will be used to address whether history of SARS-CoV-2 infection impairs systemic endothelial function and NVC response in patients with MCI. Variables included in this Aim are: NVC, band-limited power of brain waves, retinal arteriolar function, microvascular endothelial function, macrovascular endothelial function, endothelial dysfunction biomarkers, pro-inflammatory cytokine level, antioxidant capacity, COVID-19 status, SARS-CoV-2 variants, time since infection, and vaccination history.
■ Aim 2 variables will be used to address whether MCI patients with history of COVID-19 have impaired fluid cognitive function compared to MCI patients with no COVID-19 history. Variables included in this Aim are: *n*-back working memory performance, fluid intelligence, COVID-19 status, SARS-CoV-2 variants, time since infection, and vaccination history.
■ Aim 3 variables will be used to address whether history of COVID-19 in patients with previously diagnosed MCI contributes to progression to dementia, and whether impaired NVC associates with increased progression to dementia. Variables included in this Aim are: NVC, retinal arteriolar function, band limited power of brain waves, COVID-19 status, SARS-CoV-2 variants, time since infection, vaccination history, and progression of MCI to dementia.
■ Aim 4 variables will be used to address whether history of COVID-19 in MCI contributes to increased burden of WMH, and whether elevated level of WMH burden contributes to increased progression of MCI to dementia. Variables included in this Aim are: Fazekas scale, COVID-19 status, SARS-CoV-2 variants, time since infection, vaccination history, and progression of MCI to dementia.

### 2.10 Data collection process

Patients who have consented to the study are interviewed for demographic information (sex, age, race education), COVID-19 history (symptoms during infection, and symptoms without confirmed infection), COVID-19 vaccine history, medical history (oncological, genitourinary, gastrointestinal, respiratory, endocrine, neurological, cardiovascular, musculoskeletal, and psychological conditions), screened for DVA (eye surgery history, photosensitive epilepsy, glaucoma, allergies to tropicamide), and social history (previously or current use of tobacco, alcohol, and recreational drugs, and adverse childhood experiences). Medication list is obtained from all patients, and common age-related conditions (i.e., hypertension, diabetes) must be pharmacologically controlled. All patients enrolled have baseline MRI data with neuroradiologic report at MCI diagnosis. Every 6 months patients have repeat neurocognitive screening and evaluation through OUHSC Department of Neurology faculty to identify if patients have progressed from MCI to dementia. Physiologic, circulating factors, and cognitive data is obtained from patients at visit one and at 18-month follow-up. Patients are scheduled through the Department of Neurology for a second MRI to obtain neuroradiologic report at 18-month follow-up after visit one.

## 3. Methodology and data analysis

### 3.1 Cognition and neuroimaging investigations

#### 3.1.1 NIH-toolbox cognitive battery and emotional battery

Comprehensive neuropsychological testing is the gold standard for diagnosis of MCI, consisting of hours of testing to evaluate specific cognitive domains that can help identify the etiology of impairment, as well as socioeconomic and demographic factors that may contribute to cognitive decline [46]. Therefore, NIH-toolbox Cognitive Battery and select questionnaires from NIH-toolbox Emotion Battery will be implemented to assess categories of crystalized intelligence (dependent upon past learning experience), fluid abilities (domains of executive function, attention, episodic, memory, visual learning, processing speed, and working memory) and affective function. Total duration of both Cognitive and Emotion Battery is ~45 minutes and is administered at visit one and 18-month follow-up. The NIH Toolbox is a sensitive approach to evaluate age-related changes in cognitive performance, mild cognitive impairment and dementia [47], and is suitable for clinical studies with repeating measures of 4-week interval or longer [48,49]. All neurocognitive assessment will be done under supervision of a clinical neuropsychologist in the Department of Psychiatry and Behavioral Sciences at the OUHSC.

Fully adjusted scale score (mean = 50; SD = 10), which compares the score of the participant to nationally representative normative sample of NIH tool box test takers, accounting for age, sex, race, and education will be used for analysis of specific cognitive domains (picture vocabulary test, flanker inhibitory control and attention test, list sorting working memory test, dimensional change card sort test, pattern comparison processing speed test, picture sequence memory test, oral reading recognition test), and composite scores (fluid composite, crystallized composite, total composite, early childhood composite). Raw score will be used for oral symbol digit test (number of correct responses in two minutes) and unadjusted scale score (mean = 100; SD = 15) representative of normative sample without correction for other demographic variables will be used for all questionnaires within the Emotional Battery (emotional support, friendship, loneliness, perceived hostility, perceived rejection).

#### 3.1.2 Neuroimaging investigations

Prior to visit one, patients were diagnosed with MCI and neuroradiologically (i.e., MRI) assessed for small vessel complications. In a recent meta-analysis from Guo and Shi, small vessel ischemic damage to the brain, evidenced by white matter hyperintensities (WMH) on MRI, was associated with increased risk of development of MCI and dementia [50]. Ischemia localized to, and originating from small vessels, and microbleeds are two common pathologies in cerebral small vessel disease (CSVD) [51], and can be imaged by MRI through T2 fluid attenuated inversion recovery (FLAIR) and T2* gradient echo (GRE) or susceptibility weighted imaging sequences (SWI), respectively. All study participants, in addition to the first MRI scan performed as a point of care before enrollment into the study, will be neuroradiologically evaluated at Visit 2 using a 3T MRI. Increased burden of WMH (i.e., confluence of deep foci and extension of periventricular WMH into the deep white matter) are one of the most common manifestations on T2 FLAIR MRI imaging for diagnosis of CSVD [52] and is associated with acute and recovered COVID-19 patients [22]. Moreover, burden of WMH, determined by Fazekas scale [53], will be longitudinally assessed as a contributing factor for conversion of MCI to dementia in COVID-19 patients. The Fazekas scale grades the periventricular and deep white matter from 0–3, depending on the size and confluence of the lesions [53,54]. This grading system is the most widely used system to date to identify severity of white matter pathology [53,55]. Additionally, presence of microbleeds will be neuroradiologically assessed, as this pathology is also associated with COVID-19 and has a specific pattern of distribution that distinguishes CSVD burden in recovered and acute COVID-19 patients from other known causes (i.e., hypertension, acute respiratory distress syndrome) [22].

WMH burden will be evaluated at baseline by Fazekas scale to determine if there are differences at diagnosis that could influence results at cross-sectional cognitive and physiological measurements. Longitudinally, WMH burden will be calculated at 18-months to determine the influence of WMH on progression to dementia and whether COVID-19 increases burden of WMH in patients with MCI.

### 3.2 Physiological investigations

#### 3.2.1 Blood pressure measurement

Blood pressure (BP) is measured in accordance with the 2017 AHA/ACC Hypertension Guideline for Standard Measurement of BP [56]. Recordings are taken several times during each ~four-hour visit, before cognitive testing, before FMD, and before LSCI. Uncontrolled hypertension is known to cause deleterious effects to the endothelium and systemic vasculature [57]. Hence, patients with uncontrolled hypertension are excluded, in brief, due to known impairments of cerebral autoregulation [58], risk of cerebrovascular complications [59], and dysregulation of the macro- and microvasculature [60].

Systolic, diastolic, and mean arterial pressure will be calculated from patient blood pressure recordings and compared between groups (COVID-19+ and COVID-19-) to assess for differences, which may influence vascular physiological measurements.

#### 3.2.2 Functional near-infrared spectroscopy (fNIRS) and n-back paradigm

We are using fNIRS to evaluate NVC in a real-time, continuous measure, during blocks of increasing cognitive workload (*n*-back). The cognitive *n*-back is a working memory paradigm used to evoke NVC responses. Patients are seated in-front of a 22” monitor that displays the task, while they rest their right hand on the mouse. Before the measurement, patients are instructed to perform to the best of their ability and are walked through each *n*-back ‘block’ to ensure understanding of the paradigm. Thirty-two letters are presented in a randomized sequence during each block by the ePrime 3 software (Psychology Software Tools, Sharpsburg, PA) [28]. Each letter is displayed on the screen for 250 ms followed by a random time interval pause (1950±100 ms) before the appearance of the next letter. Patients are instructed to provide a correct response by click the left mouse button only when they identify a target stimulus, which depends on the type of the task for a given block. Below are the three tasks the patients are presented with:

- 0-back: identification of and response to the letter ‘**W**’. The 0-back is the first and third block of the *n*-back paradigm, with each block being a different sequence of letters with the ‘**W**’ stimulus in a randomized order.
- 1-back: identification and response to repeated letters in a sequence (e.g., Y-M-S-**S**). The target stimulus is the second letter in the sequence.
- 2-back: identification of a pattern where every other letter is repeated (e.g., Y-M-**Y**-S). The response is correct if the patient clicks the left mouse button when they recognize a letter that is the same from two positions back.

The length of each block is 72 seconds with ten second intervals between them and are administered as follows: 0-back→ 1-back→ 0-back→ 2-back.

During the *n*-back paradigm, patients wear a 128 port Easycap head cap (Easycap GmbH, Woerthsee-Etterschlag, Germany) suited with 16 sources and 16 detectors (NIRx Medical Technologies LLC, Glen Head, NY) to measure continuous changes in oxy- (HbO) and deoxy-hemoglobin (HbR) concentration. Hemodynamic responses are recorded from 48 channels (source-detector pairs) according to the international 10–20 system at a sampling frequency of 3.9 Hz and measurements are performed in a quiet, dark room. The light sources are in the NIR range, emitting light that can readily penetrate superficial layers of the scalp and skull to enter the cerebral cortex [61]. NIR wavelengths used for in vivo optical imaging are 760 nm and 850 nm, each specifically optimized for calculating HbR and HbO relative concentration changes, respectively. HbR and HbO are chromophores and based on attenuation of the NIR light at detectors, changes in hemoglobin concentration can be measured according to the continuous-wave principle [40]. These measures are limited to the cerebral cortex, a depth of 1.5 cm [62], and are standardized topographically by separating the distance between sources and detectors at 3 cm, allowing for a predictable ‘banana’ shaped path between optodes [63]. However, due to biological membranes and lipid bilayers, there is significant scattering of light in biological tissues [64]; hence, absorption changes in HbO and HbR can only be measured by the applied continuous-wave technique if constant scattering loss is assumed. To account for this, the Beer-Lambert Law (i.e., absorbance proportional to path-length and the concentration of chromophores) is modified for a highly scattering medium [65,66]. In addition to the 3 cm separated sources and detectors, channels with a smaller distance between sources and detectors (8 mm) are added to account for shallower hemodynamic changes in extracerebral structures (e.g., skin, skull), allowing for removal of extracerebral hemodynamic fluctuations [40,67]. Measurement of NVC using fNIRS is validated by functional magnetic resonance imaging (fMRI) [68] studies and is sensitive to changes in cognitive function in aging [40] and age-related diseases [42]. Additionally, decreases in HbO measured by fNIRS have been reported in mild cognitive impairment [69] and dementia [70].

fNIRS data processing and analysis will be implemented in Matlab 2022a (Mathworks, Natick, MA, USA) using custom scripts written by PM, implemented in a pipeline created by the Brain AnalyzIR toolbox (commit 46c645d) [71]. Before analysis, preprocessing steps will be applied to remove artifacts not originating from brain activation by low-pass filtering (0.02–0.4 Hz). Further, once optical densities of brain regions are transformed into hemodynamic signals by the modified Beer-Lambert law, obtained relative HbO concentrations will be pre-whitened, attenuating correlated effects due to systemic physiology and motion. Slow drifts will then be eliminated using discrete cosine transform-based high-pass filter (0.009 Hz). Scalp hemodynamic changes, captured by short channel source-detector pairs, will be subtracted from the remaining channels.

Cognitive performance during the *n*-back test will be calculated as percentage of correct responses and reaction time for each patient during each *n*-back block (0-back, 1-back, 2-back).

#### 3.2.3 Electroencephalography (EEG) and n-back paradigm

In this study, we use electroencephalography (EEG) to assess brain activity during a working memory paradigm. Like the fNIRS working memory paradigm, patients are administered an *n*-back task during EEG measurements; however, instead of letters, patients identify sequences of numbers and are instructed to click the space bar with their right hand when the stimulus appears. For 0-back, patients are instructed to hit the space bar when they see the number ‘**8**’. For 1-back and 2-back, whenever patients sees two numbers in a row or a pattern where the presented number matches the one shown before the previous number, they are expected to hit the spacebar as a correct response. EEG *n*-back was generated using a custom script created by PM, implemented in Matlab.

To measure real-time, continuous changes in brain activity, we use a wireless EMOTIV EPOC X headset (EMOTIV Inc., San Francisco, CA, USA) fit with 14 channels and 2 references, saline-based electrodes, and topography according to the international 10–20 system. The EPOC X headset has a sampling rate of 128 Hz, and bandwidth of 0.2–45 Hz with digital notch filters at 50 Hz and 60 Hz. EEG measurements are recorded with EMOTIV PRO software (EMOTIV Inc., San Francisco, CA, USA), outputting raw files that are used for further data processing, creating band-limited power spectra, averaged for each *n*- back block. Previous study has indicated changes in Beta power as being able to classify groups (AD vs. MCI vs. control) and subgroups within MCI that are likely to progress to dementia [72]. Hence, the addition of this methodology will aid in uncovering COVID-19 related changes within the neurovascular unit and better characterize the hemodynamic response measured by fNIRS. However, as COVID-19 is known as an endothelial disease [73] and data from CSF and autopsy studies indicate that neurons are likely not directly affected [74] even in olfactory impairments associated with the disease [75] we anticipate the EEG measure, in conjunction with our large battery of vascular measurements, to support that the changes in the hemodynamic response are primarily due to endothelial dysfunction.

EEG data processing and analysis will be implemented in Matlab 2022a (Mathworks, Natick, MA, USA) using custom scripts written by PM, implemented in a pipeline created by the EEGLAB toolbox [76]. Before analysis, data is cut into segments corresponding to the n-back blocks (0-back, 1-back, 2-back) and is low pass (0.5 Hz) and high pass (45 Hz) filtered. Channel locations will be defined according to the International 10–20 system. Components related to brain activity will be selected for further data analysis following wavelet-enhanced independent component analysis and multiple artefact rejection algorithm [77] aimed to attenuate extracerebral contributions to the recorded signal such as muscle activity and eye movements. Further, for each *n*-back segment, band-limited power in frequency domains of delta, theta, alpha, beta, and gamma will be generated through spectral analysis.

#### 3.2.4 Dynamic retinal vessel analysis (DVA)

The use of DVA is an emerging tool to study microvascular function in the retina, and importantly, can be used to study cerebromicrovascular pathology [78] because the retina shares embryologic origin with the central nervous system [79]. Before performing DVA, participants undergo extensive medical history screening (e.g., photosensitive epilepsy, history of glaucoma or glaucoma attack), are examined for visual acuity using a Snellen chart in a well-lit room, relevant ocular pathologies using a slit-lamp and IOP with an iCARE CI100 tonometer (Icare, Vantaa, Finland) and Goldman applanation tonometer. Chronically elevated IOP above 21 mmHg has significant risk for optic nerve damage [80] and because pupillary dilation may acutely increase IOP [81], patients are excluded from DVA measurement above an IOP of 21 mmHg. DVA is performed on the right eye after dilating the pupil with tropicamide (1% Tropicamide Ophthalmic Solution USP, AKORN, Lake Forest, IL), followed by a period of rest to ensure stable hemodynamic conditions. Using the Dynamic Vessel Analyzer (DVA, IMEDOS, Jena, Germany), the retinal arteriolar and venular diameters are evaluated [82]. Vessel segments of approximately 0.5 cm in length, located in the upper temporal quadrant at least 1 but not more than 3 papilla diameter distance from the edge of papilla, are assessed in the arteriolar and venular branches, while sites where two vessels are close to each other are avoided. The device allows for noninvasive and continuous assessment of retinal vessel diameters along a selected vessel segment. A 50 second (s) baseline recording is measured, then the first flickering stimulus (flickering light stimulation of 20 s in duration) is administered. Three flickering stimuli are administered throughout the duration of the test with 80 s of recording between stimuli. The mean of three consecutive examinations (20 s flickering stimuli and 80 s post-stimuli) are calculated for each participant [41] and used for analyses parameters. This methodology serves as a NVC measurement [41] and as a proxy for cerebromicrovascular function [79]. Hence, measured deficits in retinal arteriolar reactivity are reported to associate with cognitive impairment [78].

Data output from the retinal vessel analysis software generates continuous data for the entire 350 s measurement. With these data, we will assess relative maximal dilation percentage, relative maximal constriction percentage, time of maximal vessel dilation during flickering light stimulation, time of maximal vessel constriction, the area under the reaction curve during flickering light stimulation, and the area under the reaction curve after flickering light stimulation to determine vessel behavior following stimulus cessation.

#### 3.2.5 Assessment of peripheral macrovascular endothelial function using ultrasonography and flow-mediated dilation (FMD) protocol

To measure macrovascular endothelial function, we will use the gold standard flow-mediated dilation approach developed by Celermajer and colleagues [83] in the brachial artery (FMD) in accordance with the up-to-date tutorial presented by Harris et al. [84]. This method is endothelium dependent by opening of shear stress induced calcium channels activating endothelial nitric oxide synthase (eNOS), producing nitric oxide (NO), and allowing for diffusion of NO to the vascular smooth muscle to induce vasorelaxation. This mechanism has been validated in animal models, and in humans, showing an attenuation of the FMD response following the use of NO antagonists and an abolishment of the FMD response without an intact endothelium [85]. In addition, the use of FMD as a reliable non-invasive method to measure endothelial dependent function correlates with invasively assessed endothelial function [86]. A three lead ECG is placed on patients for continuous heart rate monitoring, allowing for removal of variability across the cardiac cycle during data analysis. Once attached, the study participants lay supine and rest for 20 minutes prior to the FMD measurement. Before baseline recording, patients’ blood pressure is measured for calculation of occlusion pressure (50 mmHg above measured systolic blood pressure, maximum 200 mHg) and a sphygmomanometer cuff is then placed below the antecubital fossa on the right forearm. The automatic cuff inflator (Hokanson E20 Rapid Cuff Inflator, Bellevue, WA, USA) is used to maintain pressure at 50 mmHg above the patients’ current systolic blood pressure during the occlusive phase. Brachial artery diameter is measured with a Doppler ultrasound system equipped with an 8–12 MHz transducer with pizo-electric crystals that transmits and receives the Doppler signal (Phillips Affinity 70, Philips North America Corporation, Cambridge MA, USA). After recording 60 s of stable baseline, FMD response is induced by release of the blood pressure cuff after 5 minutes of right forearm occlusion. After the cuff is deflated, changes in the diameter of the brachial artery are recorded for 3 minutes.

Brachial Analyzer for Research is used to calculate continuous change (15 frames per second) in brachial artery diameter across the duration of the measurement. Following calibration of the video recorded measurement, a region of interest (ROI) is selected for both baseline and post occlusion measures. ROI is selected based on signal clarity and intimal layer visibility. Following ROI selection, low boundary stiffness is selected for most precise measurement and borders determined by the BrachiAnalyzer software by automatic edge detection is accepted. Diameter measures are then calculated, and confidence threshold is set at 70%. Frames are reviewed by the operator to determine accuracy of automatic edge detection and, if needed, manual edge detection is performed to match the intimal layer. For accuracy of the baseline measurement (60 s), ten consecutive heart cycles are obtained for highest accuracy of baseline diameter. After review of all frames, auto-gating is conducted to detect R-peaks, removing influence of the cardiac cycle, resulting in end-diastolic diameter for the duration of the measurement. Processing is repeated for the post-occlusion video file. Data can then be exported, and FMD% can be used for statistical analysis [(Max diameter–average baseline diameter) / (average baseline diameter) * 100] [87].

#### 3.2.6 Assessment of peripheral microvascular endothelial function using laser speckle contrast imaging (LSCI) and flow-mediated dilation protocol

To measure endothelial function in skin microcirculation, we use the FMD approach coupled with LSCI. Decreased macro- and microvascular endothelial function is associated with cognitive dysfunction [87] and impairments in neurovascular coupling [42], leading to the hypothesis of generalized endothelial dysfunction impairing neurovascular coupling and leading to cognitive decline. This protocol is identical to the FMD approach with ultrasonography of the brachial artery; however, the blood pressure cuff is placed proximal to the antecubital fossa. Physiologic thermoregulation alters the diameter of blood vessels to either release or preserve heat [88]; therefore, to control for temperature, prior to and immediately after LSCI measurement we measure temperature of the regions of interest, middle fingernail bed and above first phalanx of middle finger, using a non-contact, laser-based thermometer (Thermoworks TW2, Thermoworks, American Fork, UT, USA). LSCI is equipped with a 785 nm laser (Perimed PSI system, Perimed, Järfälla, Sweden) and backscattering of light from movement of red blood cells through the microvasculature calculates microvascular perfusion. Through this methodology, we can measure continuous, real-time assessment of red blood cell movement in the skin microvasculature [89]. Experimental manipulations of endothelium dependent factors, such as NO, prostaglandins, adenosine and potassium, corroborates that this response is predominantly mediated by the microvascular endothelium, in conjunction with upstream mediated shear stress [90]. Similar to DVA, continuous measurement allows for multiple analyses to determine microvascular endothelial function.

Continuous measure of skin microvascular perfusion will be analyzed offline with the manufacturer’s software (PIMSoft, Perimed, Jӓrfӓlla, Sweden). We select two regions of interest, just proximal to the metacarpophalangeal joint of the middle finger, and the middle finger nailbed because this area has the highest capillary density. We will measure relative maximal dilation, time of maximal vessel dilation following release of the blood pressure cuff, and the area under the post-occlusive perfusion curve.

### 3.3 Circulating factors

Forty mL of blood will be taken from patients and centrifuged for 10 minutes at 1600 RPM or 15 minutes at 2500 RCF for separation of serum and plasma, respectively. Five, 500 μL samples of serum and plasma are aliquoted for each patient and samples are stored in a -80° freezer awaiting analysis of antioxidant capacity, cytokine levels, and endothelial biomarkers.

#### 3.3.1 Hydroxyl radical antioxidant capacity assay (HORAC)

Reactive oxygen species (ROS) have deleterious effects on the vascular endothelium [91]. Hence, antioxidant capacity is critical in combatting these insults and maintaining a healthy endothelium. Clinical and preclinical models have demonstrated that aging impairs NVC, and with the aid of antioxidant agents, can rescue NVC response [37] and improve cognition [92]. Importantly, elevations in oxidative stress are associated with patients who have recovered from COVID-19 and correlate with neuropsychiatric impairment [93]. Specifically, a recent meta-analysis showed an elevated oxidative stress/antioxidant ratio in MCI patients compared to healthy controls [94] and elevation of peripheral markers of oxidative stress in MCI, AD and VaD have been reported [95].

The antioxidant capacity of the serum samples will be measured using the Hydroxyl Radical Antioxidant Capacity (HORAC) Assay (Cell Biolabs, Inc., San Diego, CA). This 96-well plate-based assay contains a fluorescent probe which is quenched by hydroxyl radicals generated by the addition of a hydroxyl radical initiator and fenton reagent to the samples. The antioxidants present in the sample will neutralize the hydroxyl radicals and prolong the life of the fluorescent probe. Hence, more the antioxidants in the sample, the longer it takes for the complete decay of the fluorescent probe. Briefly, 20ul of the gallic acid standards or samples are added to the wells, followed by addition of 1X fluorescein solution and incubation at room temperature for 30 minutes. Then, hydroxyl radical initiator and the fenton reagent are added to the wells. After briefly shaking the plates for uniform mixing, the fluorescence will be measured in increments of 5 minutes for 1 hour using Tecan Spark multimode microplate reader. The fluorescence decay curve, represented as the area under the curve (AUC) is compared against the standard gallic acid antioxidant curve to calculate the antioxidant capacity of the serum samples fluorometrically. This measure has been previously validated in serum of patients with age-related diseases and correlates to functional measures [96].

#### 3.3.2 Magnetic bead-based multiplex assay

Circulating inflammatory cytokines and biomarkers of endothelial dysfunction present in the sera and plasma will be measured by Bio-Plex 200 System magnetic bead-based multiplex assay (Bio Rad, CA). A Milliplex Human Adipokine Magnetic Bead Kit (EMD Millipore, Billerica, MA) will be used to determine cytokines of interest: Tumor necrosis factor alpha (TNF-alpha), interleukin-1 beta (IL-1b), interleukin-6 (IL-6), interleukin-8 (IL-8) and interleukin-10 (IL-10). Multiple research groups have demonstrated that COVID-19 is associated with long-lasting elevations in inflammation that persist for at least 10-months after infection [97–99]. Importantly, elevations in pro-inflammatory cytokines are associated with systemic endothelial dysfunction in aging [100] and dementia [101]. A Milliplex Human Cardiovascular Disease Panel 1 Kit (EMD Millipore, Billerica, MA) will be used for myeloperoxidase (MPO), matrix metallopeptidase 9 (MMP-9), E selectin, vascular cell adhesion molecule-1 (VCAM-1), intercellular cell adhesion molecule-1 (ICAM-1), and plasminogen activity inhibitor-1 (PAI-1). Immunofluorescence signals will be quantified (pg/ml) and compared between groups. Growing evidence points towards endothelial impairment as a key mediator in the progression from cognitive decline to dementia. Molecules represented in the cardiovascular disease panel have been reported as biomarkers of endothelial dysfunction in vascular diseases [102] and dementia [103]. Additionally, higher levels VCAM-1 and ICAM-1 were able to differentiate AD vs MCI [104].

### 3.4 Data management

To enhance confidentiality and security, information is saved on computers that are password-protected and encrypted. Each participant is allocated an exclusive anonymous identifier that is associated with all data collected, such as questionnaires, NIH toolbox, and NVC assessment. Medical health information and demographics are collected using Redcap. Paper records, including consent form are kept in a locked, secure area that is only accessible to the research team.

### 3.5 Safety consideration

Overall, the procedures utilized in this study are associated with minimal risks that are typically associated with routine medical examinations and testing. However, there are rare potential risks involved in some procedures. For the eye examination, there is a possibility that flickering light could trigger a seizure if the participant has photosensitive epilepsy and dilating the pupils may lead to an acute glaucoma attack in patients with shallow anterior chamber depth and/or elevated intraocular pressure in rare occasions. Therefore, all participants will undergo an extensive medical questionnaire and have the eyes tested before the dilation process (eye pressure and anterior chamber depth). If history of seizures is present or high eye pressure and small anterior chamber is recorded, the participant will not be eligible for dynamic retinal vessel analysis examination. Additionally, dilation of the pupils may cause impaired vision for a few hours after the procedure, so it is advised that participants have someone drive them home on the day of their visit. As part of this study, venipuncture (blood draw) will be performed. Some risks associated with this procedure include pain from the needle insertion, discomfort from pressure and cold application (if necessary), bruising, feeling faint, and a slight risk of infection.

### 3.6 Ethical issues

The current investigation adheres to the principles outlined in the Declaration of Helsinki and has been granted ethical approval by the institutional review board (IRB) of OUHSC and VA, under protocol number 14585. Prior to participation, individuals will provide written informed consent by signing a statement of agreement. Participants are free to withdraw from the study at any time without consequence. In instances where a participant is unable to provide consent, a substitute written consent from a designated family member or caregiver will be accepted. Respect for each participant’s personal beliefs will be taken into consideration, and all individuals must provide their assent to the research protocol. Given the progressive and ongoing nature of the illness being studied, the capacity for consent may need to be periodically reevaluated, and this will be done on an as-needed basis.

### 3.7 Length of study

Participant enrollment for this study is expected to take 2 years, allowing for baseline physiological, cognitive, circulating factors, and neuroimaging data from up to 200 patients. Each patient will undergo a second visit and MRI 18-months following their baseline visit, bared an early termination or drop-out. Follow-up measurements are expected to conclude in 2026.

## 4. Statistical analysis

### 4.1 Cross-sectional data analysis

Upon testing for normality of continuous data, independent t-test or Mann-Whitney *U* Test will be used for group comparison (COVID+ vs COVID-) and Chi-square or Fisher’s exact test for group comparison of categorical variables. Effect size will be calculated for all within and between group analyses. For **primary outcome** analysis, testing whether COVID-19 impairs NVC responses in MCI patients, fNIRS data will be analyzed using a pipeline based on General Linear Model (GLM) approach created using the Brain AnalyzIR toolbox. At the first (subject) level, regression (β) coefficients characterize NVC response and will be estimated for channels of interest in the prefrontal cortex across all n-back blocks and chromophores (HbO and HbR). At the second (group) level the GLM treats COVID status as an independent categorical factor, while the n-back block as a repeated measures factor and yields corresponding *t*-contrasts comparing NVC response within and between groups. We will test whether demographic variables, COVID-19 variants, duration of COVID-19 and vaccination influences the primary outcome through linear regression. Moreover, we will create a multivariate model using β coefficients, generated from the fNIRS pipeline, to determine whether COVID-19 predicts impairment in NVC in MCI patients. Univariate analyses of factors that may contribute to NVC impairment (i.e., age, sex, baseline structural MRI abnormalities) will be conducted, and analyses with a p value of <0.2 will be included in the multivariate model. **Secondary outcome** measures (i.e., FMD, LSCI, EEG, DVA, NIH Toolbox, circulating factors) will be compared between groups. Additionally, we will determine whether history of COVID-19 contributes to impairment in functional cognitive measures (i.e., n-back, NIH Toolbox) by multiple and simple linear regression. For **exploratory analyses**, we will determine whether group (COVID+ vs COVID-) alters the relation between NVC and peripheral, cognitive, and structural measures through multiple regression. Correlations for these variables will be performed within groups to determine strength of relationship with NVC.

### 4.2 Longitudinal data analysis

Continuous data will be analyzed by 2-way ANOVA and categorical with Chi-square or Fisher’s exact test for within and between groups (COVID+ vs COVID-) comparison. Effect size will be calculated for all within and between group analyses. For **primary outcome** analysis, we will determine whether COVID-19 related impairment in NVC and increases in WMH burden contributes to progression to dementia. Progression to dementia within and between groups will be analyzed and multivariate analysis will be conducted to determine the effect of COVID-19, NVC (i.e., fNIRS), change in WMH burden, and interaction between group (COVID+, COVID-) and NVC and WMH burden on progression to dementia. **Secondary outcome** measures (i.e., FMD, LSCI, EEG, DVA, NIH Toolbox, circulating factors) will be compared within and between groups. Demographic variables, COVID-19 variants, duration of COVID-19 and vaccination will be assessed as factors for progression to dementia through logistic regression. **Exploratory analyses** will include univariate analysis of all demographics, physiologic, cognitive, and structural variables, and their effect on progression to dementia. All values with a p value <0.2 will be included in a multivariate model to assess their contribution to progression to dementia. We expect that several variables will have p value <0.2; therefore, we will conduct dimensionality reduction before adding into the model to assess variables which have strongest correlation with principal components. The limitation for the multivariate model will be sample size, however this is not a primary outcome measure and is used to generate new hypotheses to test in future studies.

## 5. Discussion

Given the high burden of COVID-19 infection and increasing number of elderly individuals in our population, this study has the potential to shed light on the burden and predictors of cognitive decline and dementia in our setting. COVID-19 patients are prone to develop cerebrovascular complications, particularly among older individuals and those with age-related conditions [22]. Although the underlying etiology of cerebrovascular accidents in this patient population are not yet fully understood, research suggest that the SARS-CoV-2 virus may have a systemic impact, increasing age-related chronic elevation in thromboinflammation and reactive oxygen species (ROS) levels, which can have detrimental effects on the vasculature [93,97–99,105,106]. Previous studies have shown that impairment of NVC and endothelial function play a significant role in the pathogenesis of cognitive decline and dementia [43] and there is a growing body of evidence linking COVID-19 with endothelial dysfunction [106,107]. Cognitive impairment has been identified as a significant complication following recovery of acute COVID-19 [108]. Additionally, COVID-19-induced increases in the burden of WMH have been linked to an increased risk of dementia in patients with MCI [21]. We predict that COVID-19 infection will promote systemic endothelial dysfunction, impair NVC responses and promote white matter damage, contributing to the progression of MCI to dementia. Moreover, we expect that COVID-19 infection will lead to an elevation in circulating reactive oxygen species (ROS), proinflammatory cytokines, and biomarkers of endothelial dysfunction. Elevation in these molecules have a deleterious effect on the vasculature [91] and is implicated in the development of cognitive decline and dementia [103,109]. Furthermore, evidence suggests that following recovery of COVID-19 infection, there is a prolonged thromboinflammatory response, in part, mediated by systemic endothelial dysfunction [106].

Conditions known to cause elevations in oxidative stress and chronic inflammation (i.e., aging [110], diabetes [111], hypertension [112], dyslipidemia [113], obesity [114]) have impaired cerebromicrovascular function [32,115,116] (e.g., endothelial nitric oxide synthase uncoupling, peroxynitrite formation) and contributes to a decreased NVC response, resulting in cognitive dysfunction [16,40,41,117,118]. However, in preclinical and clinical studies, the NVC response can be rescued, improving cognitive outcomes in aging and age-related diseases by antioxidant rich treatments [33,34,37–39]. Hence, as elevations in ROS has been shown in recovered COVID-19 patients and chronically elevated oxidative stress may play a large role in persistent endothelial dysfunction [105] and long-COVID symptoms [119], such as cognitive impairment or “brain fog”, the use of antioxidant treatments may be of interest to rescue a potentially dysregulated NVC response. A recent systematic review has outlined the beneficial use of antioxidants (i.e., vitamins C, D, selenium and zinc) thus far for the treatment of COVID-19 complications [120], and vitamin C, specifically, has shown endothelial protective effects [106]. In addition, Tocilizumab, a humanized anti-IL-6 receptor antibody, has shown beneficial endothelial effects [121], potentially due to glycocalyx thickening, reducing the high inflammatory and oxidative stress burden seen in COVID-19 [106]. While antioxidant treatments have been trialed for acute COVID-19 conditions, albeit limited, the use of treatments with beneficial effects on oxidative stress levels is recovered COVID-19 patients are scarce.

Nicotinamide adenine dinucleotide (NAD+) supplementation is of particular interest in the mitigation of long-term COVID-19 symptoms. Low NAD+ levels are implicated in the pathogenesis of age-related diseases associated with poor COVID-19 outcomes. Hence elevating NAD+ levels may decrease inflammation and high levels of ROS, promoting anti-viral defense, and potentially alleviating long-term COVID-19 complications [122,123]. Nicotinamide riboside (NR) is an NAD^+^ precursor and administration of NR, or other precursors (nicotinamide mononucleotide [NMN]) restored NAD+ levels in aged cerebromicrovascular endothelial cells (CMVEC), reduced CMVEC mitochondrial ROS, and rescued NVC responses in old mice, associating with improved cognitive performance [38]. NAD+ is a rate limiting co-substrate for sirtuin enzymes, which are involved in maintaining proper mitochondrial function (e.g., mitophagy, biogenesis) [124], and protect the cell from aberrant ROS production [125]. In addition, pharmacological SIRT1 activator SS-31 improved cerebromicrovascular endothelial function, NVC responses, and cognitive function, further indicating a role for NAD+ and NAD+ intermediates to be translated from preclinical to clinical trials [39]. NAD+ bioavailability decreases as we age and during viral infections, in part due to upregulation of NAD+ consuming enzymes, such as poly(ADP-ribose) polymerase 1 (PARP1) [122], which interacts with NF-kB signaling, precipitating further chronic inflammation and oxidative stress [91]. Moreover, we are currently conducting a clinical trial to elucidate the effects of NR supplementation on systemic endothelial function, NVC responses, and cognition in healthy aging individuals (Effect of NAD supplementation on brain vascular health in aging) with the anticipation of identifying cognitive and vascular benefits reported in preclinical trails. Following this, NR supplementation may prove beneficial in future trials targeting cerebromicrovascular endothelial dysfunction in age related diseases and conditions known to deplete NAD+. NAD+ intermediate supplements may provide relief from persistent COVID-19 related cognitive dysfunction and need to be investigated as a therapeutic to target the NVC mechanism.

As with any longitudinal observational study, maintaining contact with all participants can be difficult, particularly when dealing with extended follow-up periods. Losing contact with a significant number of patients can result in biased outcomes, especially if the reason for loss to follow-up is related to the risk factor or outcome of interest—dementia. Additionally, due to the presence of confounding variables, observational studies have limited capacity to establish causation from the observed association between COVID-19 infection and conversion rate to dementia. Therefore, it is necessary to distinguish the effects of COVID-19 from other factors such as medical history, comorbidities, hospital interventions, and critical care status. In conclusion, cognitive impairment is linked to COVID-19, and it is a major contributor small vessel cerebrovascular complication. Therefore, we will elucidate a mechanism for how history of COVID-19 affects cognitive function and can pave the way for future therapeutic interventions targeting neurovascular uncoupling as a prominent feature in the conversion to dementia.
